# Supplementary material for: Prediction of SARS-CoV-2-positivity from million-scale complete blood counts using machine learning
Source: Commun Med (Lond). 2022 Jun 15;2:72. doi: 10.1038/s43856-022-00129-0 (PMC9199341; doi:10.1038/s43856-022-00129-0)
Supplement: Supplementary file 1 — Supplementary Information [file 43856_2022_129_MOESM1_ESM.pdf]

Learning COVID-19 virus-specific diagnostic models from million-scale complete blood counts

Supplementary Information

Gianlucca Zuin[1,2], Daniella Araujo[1,2], Vinicius Ribeiro[2], Maria Gabriella Seiler[2], Wesley Heleno Prieto[3], Maria Carolina Pintão[3], Carolina dos Santos Lazari[3], Celso Francisco Hernandes Granato[3], and Adriano Veloso[1]

[1]Universidade Federal de Minas Gerais, Computer Science Department, Belo Horizonte, Brazil
[2]Kunumi, Belo Horizonte, Brazil
[3]Grupo Fleury, São Paulo, Brazil

Supplementary Figures

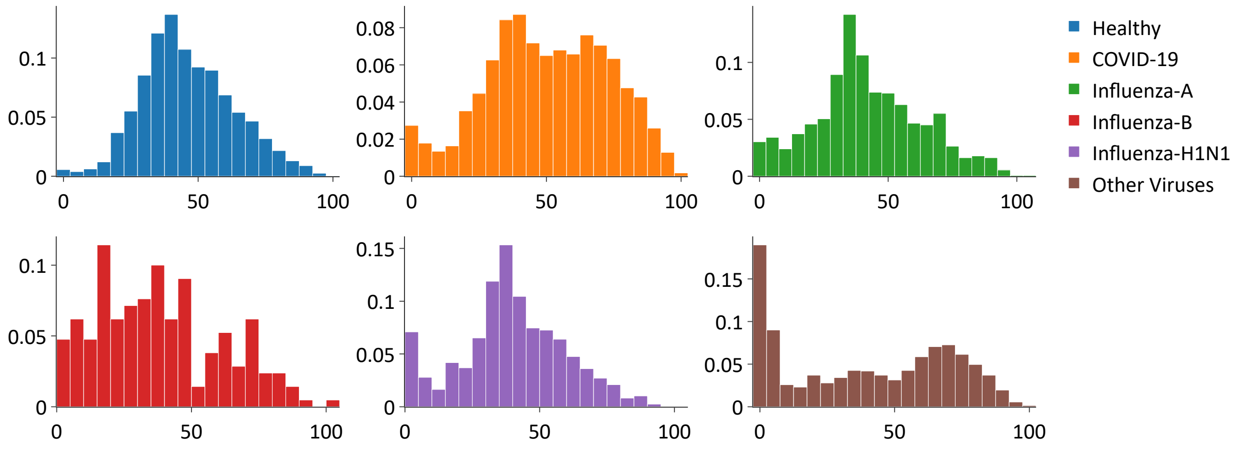

Supplementary Figure 1: Dataset age distribution. Age distribution of the patients across all the evaluated diseases. Only patients with a positive RT-PCR were considered. Number of patients available on Table 1.

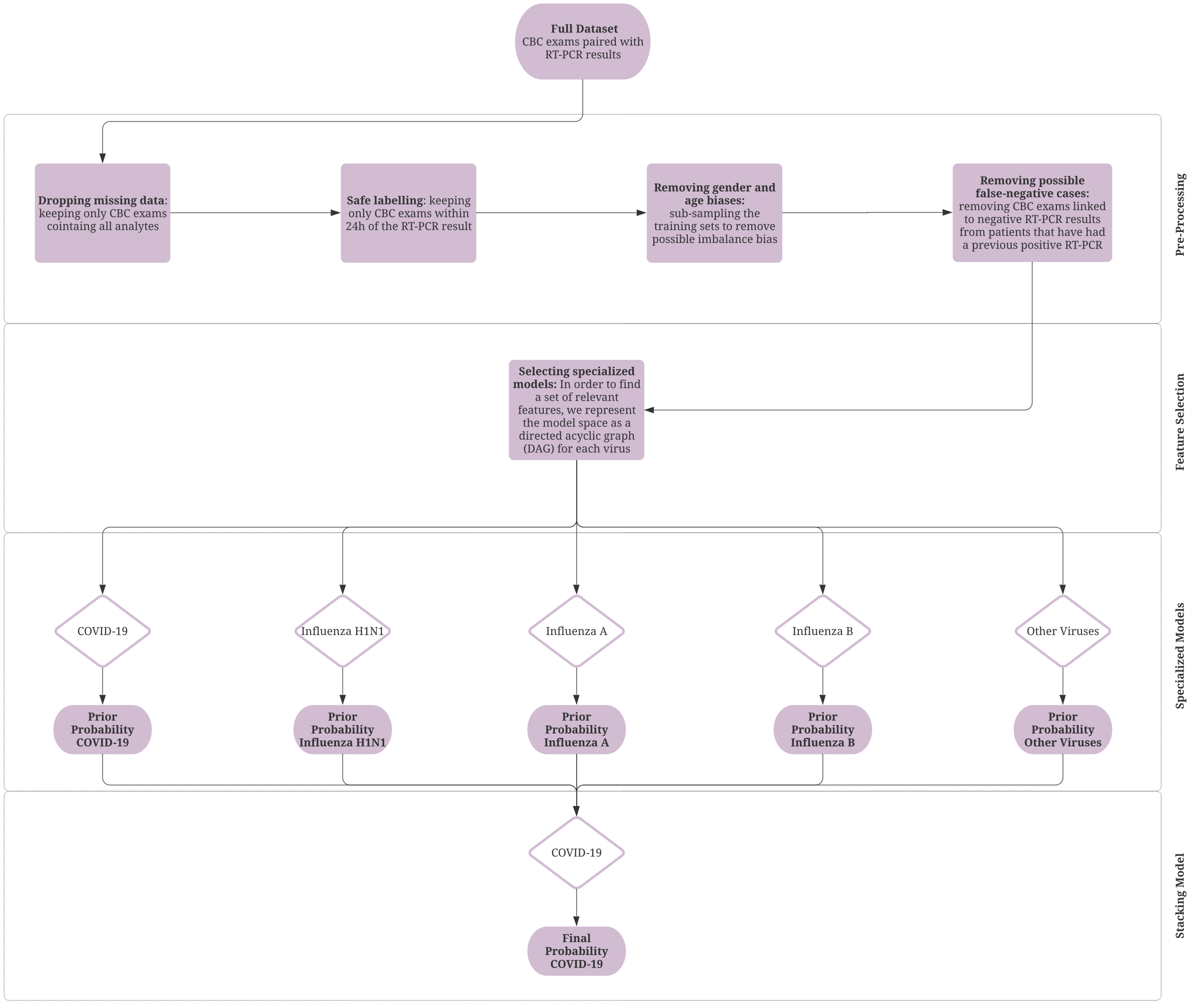

Supplementary Figure 2: Flowchart of the proposed learning approach. The proposed pipeline can be divided into four steps. In the first one, we remove biases and missing data, while also only retaining the exams in which we have confidence in the RT-PCR result. The second step constitutes searching the $N!$ space through the set of optimal features. In the third step, we train specialized base models onto each of the proposed target diseases. The final step constitutes learning a stacking model that combines the outputs of each specialized base model onto the final probability score.

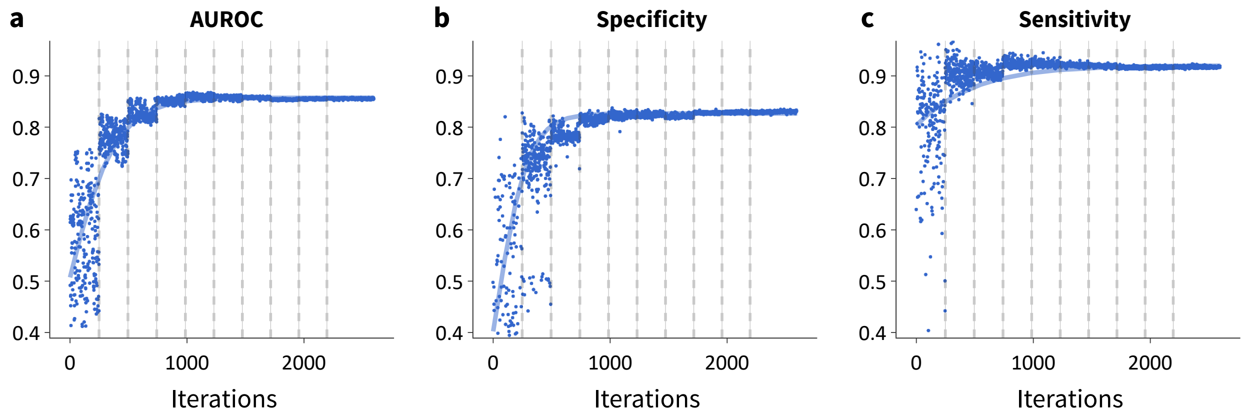

Supplementary Figure 3: Performance during feature selection greedy search. Increase in performance as we allow more features to enter the model while performing a greedy search (i.e., each iteration increases the feature size). Each point in the figure represents a COVID-19 model, and the number of features within a model is given according to the corresponding dashed lines. **a** Performance increase of the area under the Receiver operating characteristic curve. **b** Performance increase of specificity. **c** Performance increase of sensitivity.

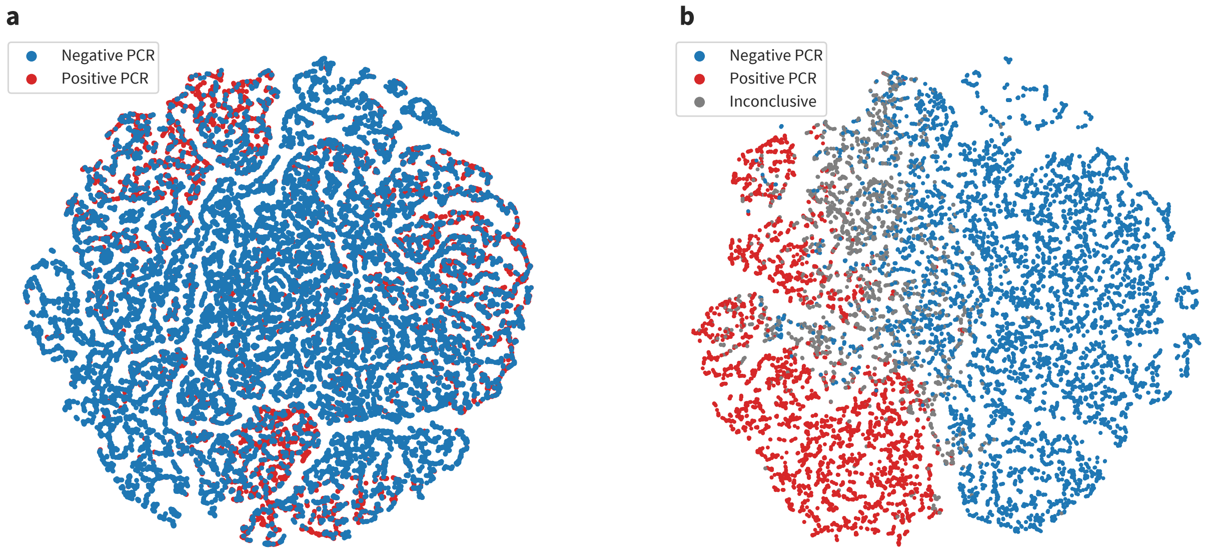

Supplementary Figure 4: Representation of the CBC space. **a** TSNE representation of the CBC space using analyte's raw data. **b** TSNE representation of the CBC space using analyte's shapley values and model predictions.
