# Supplementary material for: Prediction of SARS-CoV-2-positivity from million-scale complete blood counts using machine learning
Source: Commun Med (Lond). 2022 Jun 15;2:72. doi: 10.1038/s43856-022-00129-0 (PMC9199341; doi:10.1038/s43856-022-00129-0)
Supplement: Supplementary file 3 — Reporting Summary [file 43856_2022_129_MOESM3_ESM.pdf]

nature portfolio

Corresponding author(s): Gianlucca Lodron Zuin

Last updated by author(s): May 19, 2022

Reporting Summary

Nature Portfolio wishes to improve the reproducibility of the work that we publish. This form provides structure for consistency and transparency in reporting. For further information on Nature Portfolio policies, see our Editorial Policies and the Editorial Policy Checklist.

Statistics

For all statistical analyses, confirm that the following items are present in the figure legend, table legend, main text, or Methods section.

n/a	Confirmed	
☐	☒	The exact sample size (n) for each experimental group/condition, given as a discrete number and unit of measurement
☐	☒	A statement on whether measurements were taken from distinct samples or whether the same sample was measured repeatedly
☐	☒	The statistical test(s) used AND whether they are one- or two-sided<br>*Only common tests should be described solely by name; describe more complex techniques in the Methods section.*
☒	☐	A description of all covariates tested
☒	☐	A description of any assumptions or corrections, such as tests of normality and adjustment for multiple comparisons
☐	☒	A full description of the statistical parameters including central tendency (e.g. means) or other basic estimates (e.g. regression coefficient) AND variation (e.g. standard deviation) or associated estimates of uncertainty (e.g. confidence intervals)
☒	☐	For null hypothesis testing, the test statistic (e.g. F, t, r) with confidence intervals, effect sizes, degrees of freedom and P value noted<br>*Give P values as exact values whenever suitable.*
☒	☐	For Bayesian analysis, information on the choice of priors and Markov chain Monte Carlo settings
☐	☒	For hierarchical and complex designs, identification of the appropriate level for tests and full reporting of outcomes
☒	☐	Estimates of effect sizes (e.g. Cohen's d, Pearson's r), indicating how they were calculated

Our web collection on statistics for biologists contains articles on many of the points above.

Software and code

Policy information about availability of computer code

Data collection	InterSystems Caché and Ensemble, version 1.4, stored in a Microsoft SQL format.
Data analysis	Python 3.7, employing the packages scikit-learn v0.24, SciPy v1.6.0 and SHAP v0.37<br><br>The code used for the machine-learning analyses available for non-commercial use has been deposited at https://doi.org/10.6084/m9.figshare.15046797

For manuscripts utilizing custom algorithms or software that are central to the research but not yet described in published literature, software must be made available to editors and reviewers. We strongly encourage code deposition in a community repository (e.g. GitHub). See the Nature Portfolio guidelines for submitting code & software for further information.

Data

Policy information about availability of data

All manuscripts must include a data availability statement. This statement should provide the following information, where applicable:

- Accession codes, unique identifiers, or web links for publicly available datasets
- A description of any restrictions on data availability
- For clinical datasets or third party data, please ensure that the statement adheres to our policy

Data Availability Statement
All source data for the figures in the main manuscript and the Supplementary Information available for non-commercial use have been deposited at https://doi.org/10.6084/m9.figshare.15046797. Additional datasets were used in this study under specific conditions and are restricted due to confidentiality limitations. Requests to access additional datasets should be directed to wesley.prieto@grupofleury.com.br and will undergo internal approval from Grupo Fleury.

Code Availability Statement
The code used for the machine-learning analyses available for non-commercial use has been deposited at https://doi.org/10.6084/m9.figshare.15046797.

Field-specific reporting

Please select the one below that is the best fit for your research. If you are not sure, read the appropriate sections before making your selection.

☒ Life sciences ☐ Behavioural & social sciences ☐ Ecological, evolutionary & environmental sciences

For a reference copy of the document with all sections, see nature.com/documents/nr-reporting-summary-flat.pdf

Life sciences study design

All studies must disclose on these points even when the disclosure is negative.

Sample size	Our working dataset consisted of data from almost 1 million patients from all regions of Brazil, encompassing more than 2 million exams. For validation alone, we work with more than 300.000 complete blood counts (CBC) following the exclusion criteria mentioned below.
Data exclusions	We exclude from our analysis instances that were taken far from the acquisition date of the corresponding RT-PCR to obtain safe labels for each exam. We also remove probable False-Negative results that were interspersed with two or more positive results. Finally, to remove gender bias, we undersample the Training set to retain an even male to female ratio.
Replication	The paper fully describes the steps needed to replicate our experiments. That is: collect complete blood counts (CBCs) and RT-PCR for a large number of patients, including both COVID-19 and other pathologies; explore the model space by greedy feature addition to reach a performant model; plot analyte importance and evaluate the trained model on the specified scenarios.
Randomization	We employed the stratified k-fold algorithm of scikit-learn and numpy random number generator to randomize patient samples for cross-validation and obtain statistical significance. Thus, we guarantee that different exams from the same patient remain in the same fold avoiding data leakage.
Blinding	All the exams were anonymized prior to the conduction of the research.

Reporting for specific materials, systems and methods

We require information from authors about some types of materials, experimental systems and methods used in many studies. Here, indicate whether each material, system or method listed is relevant to your study. If you are not sure if a list item applies to your research, read the appropriate section before selecting a response.

Materials & experimental systems

n/a	Involved in the study	
☒	☐	Antibodies
☒	☐	Eukaryotic cell lines
☒	☐	Palaeontology and archaeology
☒	☐	Animals and other organisms
☐	☒	Human research participants
☐	☒	Clinical data
☒	☐	Dual use research of concern

Methods

n/a	Involved in the study	
☒	☐	ChIP-seq
☒	☐	Flow cytometry
☒	☐	MRI-based neuroimaging

Human research participants

Policy information about studies involving human research participants

Population characteristics	1,138,728 anonymized CBC exams from 900,220 unique patients, of which 45.6% belong to a male and 54.2% belong to a female, were employed in this study. Patients are aged between 1 and 107 years old (mean 51.15 +/- 22.93 years). This work does not investigate demographic, prognostic, or clinical data, such as ethnicity, hospitalization, or symptomatology, as these fall out of laboratory scope.
Recruitment	All patients that performed a CBC and RT-PCR for COVID-19 between January 2020 and March 2021 at one of Grupo Fleury laboratories were subject in this study.
Ethics oversight	Research Ethics Committee (CEP) of Grupo Fleury (CAAE: 33790820.3.0000.5474)

Note that full information on the approval of the study protocol must also be provided in the manuscript.

Clinical data

Policy information about clinical studies

All manuscripts should comply with the ICMJE guidelines for publication of clinical research and a completed CONSORT checklist must be included with all submissions.

Clinical trial registration	No intervention was performed, this study encompasses a retrospective study of the anonymized data.
Study protocol	This project was submitted, evaluated, and approved by the Research Ethics Committee (CEP) of Grupo Fleury (CAAE: 33790820.3.0000.5474), duly qualified by the National Research Ethics Committee (CONEP) of the National Health Council of Brazil. The Research Ethics Council (CEP) is an interdisciplinary and independent collegiate of public relevance, consultative, deliberative, and of educational character, created to defend the interests of research participants in their integrity and dignity as well as to contribute research development within highest ethical standards. By decision of the CEP, since this project uses retrospective and anonymized data, there is no need to apply an e Free and Informed Consent Term (TCLE) to participating patients.
Data collection	The Fleury database structure was created on 10/1997 using an InterSystems Caché and Ensemble, version 1.4 (Caché, InterSystem, 2018), a high-performance architecture that is commonly used to develop software applications for healthcare management (Cambridge MA). The database was built using standard healthcare industry practices to ensure accuracy, completeness, and security of data collected. The results of the laboratory tests are automatically inserted in a Microsoft SQL database after verification of the RT-PCR output. Within a few seconds, data is replicated to the Cache Database - Intersytems - for permanent storage. Once stored in the database, the result is made available for patients. All users have a username and password, maintained by AD Windows (Active Directory). All registry changes to the database are tracked through a log and are restricted to users with high-level administrative permissions. Information is kept secure through a separate network firewall, accessed only by authorized persons within the Fleury Group's domains. All patients that performed a CBC and RT-PCR for COVID-19 between January 2020 and March 2021 at one of Grupo Fleury laboratories were subject in this study.
Outcomes	No intervention was performed, this study encompasses a retrospective study of the anonymized data. We compared patients that tested positive to COVID-19 after performing a RT-PCT exam to those who tested negative.
